# Modulatory effects of *Boletus edulis* on the gut microbiota in Atlantic salmon (*Salmo salar*) utilizing an artificial teleost gut model

**DOI:** 10.1186/s42523-025-00469-x

**Published:** 2025-10-27

**Authors:** Alexandru S. Barcan, Joseph L. Humble, Rares A. Barcan, Eve Hughes, Brendan Robertson, Douglas J. Morrison, Emanuel Vamanu, Philip McGinnity, Martin S. Llewellyn

**Affiliations:** 1 SBOHVM, University of Glasgow, Graham Kerr Building, Glasgow, G12 8QQ UK; 2 Maths & Physical Sciences, University of Sussex, Falmer, Brighton, BN1 9RH UK; 3 School of Biological, Earth & Environmental Sciences, University College Cork, Cork, Ireland; 4 Faculty of Biotechnology, University of Agricultural Sciences and Veterinary Medicine, 011464 Bucharest, Romania; 5 Scottish Universities Environmental Research Centre (SUERC), College of Science and Engineering, University of Glasgow, Glasgow, UK; 6 Anoom Laboratories SRL, Pipera-Tunari Road 1 (Euromaster Building), 077190 Voluntari, Romania; 7 Institute for Ocean Engineering, Shenzhen International Graduate School, Tsinghua University, Shenzhen, 518055 China

**Keywords:** *Boletus edulis*, Gut microbiota, Atlantic salmon, Prebiotics, Mushrooms, Aquaculture, Nutrient absorption

## Abstract

**Background:**

In aquaculture, several functional mushrooms have been efficiently used as prebiotics, impacting gut microbiota, increasing growth, and delivering antioxidant advantages to a variety of finfish species. However, the potential of *Boletus edulis*, the ‘porcini’ or ‘penny bun’ to influence the gut microbiota of *Salmo salar* has yet to be studied. Here, we investigated the prebiotic effect of *Boletus edulis* extract (BEE) on the gut microbiota of farmed Atlantic salmon via an in vitro gut model (SalmoSim).

**Results:**

Notable changes in the production of short-chain fatty acids and microbial diversity were observed upon the addition of BEE. In particular, increased fiber fermentation was suggested by the decreasing concentrations of ammonia and increasing levels of acetate and propionate. Moreover, the 10% BEE improved the absorption of amino acids and increased the digestibility of crude protein, promoting a more diverse microbial community and reducing the accumulation of nitrogenous waste.

**Conclusions:**

The results of the present study revealed that the addition of BEE efficiently altered the gut microbiota, increasing microbial diversity, supporting beneficial short-chain fatty acid synthesis, and improving nutritional absorption in Atlantic salmon.

**Supplementary Information:**

The online version contains supplementary material available at 10.1186/s42523-025-00469-x.

## Background

Atlantic salmon (*Salmo salar*) farming has grown from small-scale supplementation of wild stocks into a major global industry producing most of the salmon consumed worldwide [1]. In this species, digestion and nutrient absorption are closely linked to the gut microbiota, which produces short-chain fatty acids (SCFAs) that influence host metabolism and health. Similar to mammals, the gut microbiota in fish plays a central role in digestion, nutrient assimilation, and immune defense [2, 3]. It is shaped by host-specific factors and is physiologically important for growth and health [4, 5]. Because its equilibrium depends on complex interactions among many microbial species, it is one of the most dynamic and sensitive components of the fish organism [6–8]. Gut microbiota stability can be disturbed by factors such as diet composition, antibiotics, or fasting [9, 10], making nutritional interventions an important way to influence digestion and fermentation in aquaculture systems. Maintaining a stable and diverse gut microbiota is important in aquaculture because it supports efficient digestion, nutrient absorption, and balanced fermentation, processes that are central to fish growth and feed efficiency [11].

Microbial carbohydrate fermentation in the vertebrate gut typically produces SCFAs such as acetate and propionate, which support gut function, nutrient absorption, and microbial balance [12–14]. In contrast, protein fermentation leads to deamination of amino acids, resulting in branched-chain fatty acids (BCFAs) and ammonia, a toxic byproduct that reflects inefficient protein utilization [15]. These fermentation profiles are important indicators of microbiota function and diet quality, especially in low-fiber aquafeeds where microbial metabolism may shift toward proteolysis.

Prebiotic compounds are indigestible feed ingredients that help the host by selectively promoting a beneficial gut microbiota by acting as microbial growth substrates [16]. Although, prebiotics have been shown to improve animal health, their use in aquaculture is still limited, and their underlying mechanisms of action are still being investigated [17, 18]. Mushrooms are a promising source of prebiotic compounds because they contain complex polysaccharides, including β‑glucans, mannans, and chitin [19–21]. β-glucans have noteworthy prebiotic properties [22]. The bulk of polysaccharides generated from mushrooms are categorized as β-glucans, despite their varied chemical makeup [23]. In Atlantic salmon, macrophages from the anterior kidney exhibit increased phagocytic activity and superoxide production when stimulated by β-glucans, which simultaneously increase lysozyme activity within the organ, collectively enhancing the fish’s innate immune response [24, 25]. The vertebrate genome lacks the necessary genes required to synthesize digestive enzymes capable of hydrolyzing the β-glucosidic bonds found in β-glucans [26, 27]. Consequently, these polysaccharides resist acid hydrolysis in the stomach and remain indigestible as they pass through the digestive tract [28]. However, once they encounter gut microbes equipped with appropriate carbohydrate-active enzymes (CAZymes), β-glucans can be broken down, producing various metabolic byproducts that may benefit the host [29]. The inherent indigestibility of mushroom-derived carbohydrates increases their potential as prebiotics, as they can selectively stimulate beneficial microbial activity within the gut. The incorporation of prebiotics, specifically mushroom-derived products such as mycelial liquid biomass, stalk waste extracts and β-glucans, into fish diets has been shown to increase innate immune responses, provide protection against infectious diseases, improve hematological parameters, and enhance growth [30–35]. The inherent indigestibility of mushroom-derived carbohydrates increases their potential as prebiotics, as they can selectively stimulate beneficial microbial activity within the gut. The incorporation of prebiotics, specifically mushroom-derived products such as mycelial liquid biomass, stalk waste extracts and β-glucans, into fish diets has been shown to increase innate immune responses, provide protection against infectious diseases, improve hematological parameters, and enhance growth [30–35]. Diets supplemented with *Neurospora intermedia* significantly increased *Lactococcus* abundance in rainbow trout, with feeding duration playing a greater role in shaping the gut microbiota than does diet preprocessing [36]. Similarly, *Geotrichum candidum* supplementation in gibel carp enhances gut microbiota diversity, growth, and immune response, leading to improved disease resistance against *Aeromonas hydrophila* [37]. Additionally, research on sea cucumbers (*Apostichopus japonicus*) has shown that supplementation with *Cordyceps* polysaccharides significantly optimized the gut microbiota composition and increased beneficial bacteria such as *Bacillus* and *Lactobacillus* while enhancing immunity and disease resistance against *Vibrio splendidus* [38]*.* Other studies on mushrooms (such as *Ganoderma lucidum*, *Agaricus bisporus*, and *Pleurotus ostreatus)* and their extracts have shown improved growth performance and maturation across a range of fish species [39–41].

*Boletus edulis*, which is commonly known for its culinary use, has demonstrated notable gut microbial and immunomodulatory effects on the human gut microbiota by stimulating the growth of beneficial bacteria, increasing microbial diversity, and promoting the synthesis of SCFAs [42]. Its antioxidant and anti-inflammatory properties further support gut health by alleviating oxidative stress and reducing inflammation [43]. Guided by these findings, this study investigated the effects of *Boletus edulis* extract (BEE), a mushroom-derived feed additive rich in bioactive compounds, including 3.7 ± 0.5% β-glucans and 9.56% crude protein, as well as numerous polyphenols such as catechin (83.9 μg/g), rutin (311.5 μg/g), hesperidin (18.0 μg/g), and sinapic acid (22.8 μg/g) (Table 1) [42] to modulate the gut microbiota of *Salmo salar* using the artificial gut system SalmoSim [44]. The aim was to determine whether incorporating BEE in the simulated salmon diet could modulate gut microbiota composition, enhance SCFA production, reduce ammonia accumulation, and improve protein digestion and absorption in Atlantic salmon, recognizing the potential of mushroom-derived compounds as underused functional ingredients in fish diets.

**Table 1:** Composition of *Boletus edulis* extract (BEE). Values are means ± SD. Units are % (w/w) for protein and beta-glucans, and μg/g dry weight (DW) for phenolic compounds

Component	Value
Crude Protein	9.56% (w/w)
Beta-glucans	3.7 ± 0.5% g/100 g BEE powder
Phenolic Compounds (μg/g DW)	
Hesperidin	17.967 ± 0.621
Catechin	83.940 ± 0.137
Naringenin	17.077 ± 0.174
Rutin	311.476 ± 3.444
Cinnamic acid	6.829 ± 0.057
Chlorogenic acid	9.239 ± 0.167
Sinapic acid	22.798 ± 0.111
Syringic acid	8.101 ± 0.101
Ferulic acid	7.218 ± 0.044
Myricetin	3.392 ± 0.016

## Materials and methods

### Atlantic salmon gut sample collection and preparation

Gastrointestinal samples from Atlantic salmon were obtained and processed for microbial analysis. Three digestive systems were extracted from healthy, cultivated mature salmon. These samples were conveyed to the research facility in oxygen-free conditions, chilled in an anaerobic box to preserve their state and avoid bacterial infiltration. Once in the laboratory, the finger-like protrusions near the stomach (pyloric caeca) were meticulously separated from each gut sample. A one-gram portion of intestinal microbial matter, encompassing both mucous and surface scrapings from the inner lining, was removed from each cecum and placed into individual sterile freezing vials. The microbial extracts were flash frozen in liquid nitrogen, and subsequently stored at − 70 °C. Freezing at − 70 °C is a standard practice widely used to preserve microbial DNA for microbiome studies [45]. Furthermore, this method is considered reliable for maintaining DNA integrity and minimizing changes in microbial community composition, making it suitable for analyzing alpha and beta diversity scores [46, 47]. While freezing halts bacterial metabolic activity, it preserves DNA profiles of most taxa for subsequent 16S rRNA gene sequencing. It should be noted, however, that functional activity (e.g., metabolism) of bacteria is not well preserved under these conditions [48, 49].

### Preparation of BEE

The preparation of the BEE was previously described in [42]. In summary, a two-step extraction was performed. The first step involved hot water extraction to isolate the polysaccharide-rich fraction. In the second step, the residue was diluted 1:1 with water, and after 2 h, ethanol was added gradually to reach a final concentration of 50% (w/v). Viscozyme L was included during this ethanol extraction step to enhance the release of polyphenolic and carotenoid compounds.

The mixture was left for up to 24 h, then filtered, and ethanol was removed by evaporation. The resulting ethanol-free fraction was combined with the aqueous extract from the first phase. To facilitate spray drying, 7% (w/v) Maltodextrin and 3% (w/v) green barley powder were added as carrier agents. The β-glucan content of the final extract was quantified using the Megazyme β‑Glucan Assay Kit (Megazyme International, Ireland), following the manufacturer’s instructions. The composition of the resulting BEE is summarized in Table 1.

### Fish feed medium preparation

The fish feed used in the experiment was purchased from EWOS HARMONY and had the following composition (Table 2).

**Table 2:** Nutritional composition of control fish feed used in the experiment

Analytical constituents	%
Oil	27.4
Ash	7.4
Fiber	1.5
Protein	41.1
Calcium	1.3
Phosphorus	1.1
Sodium	0.6

To formulate the feed medium, a Duran bottle was filled with 2 L of Milli-Q water, into which 70 g of Instant Ocean and 20 g of pulverized fish pellets (previously ground into a fine powder using a blender) were introduced. To mimic the enzymatic breakdown occurring in a salmon stomach, the acidity of the solution was increased to pH 3.5 using 1.0 M hydrochloric acid (HCl), added dropwise while monitoring with a calibrated pH meter followed by the addition of 12 ml salmon enzymes under constant agitation. This enzymatic digestion process was Maintained for 60 min at approximately 12 °C, Matching the physiological temperature of Atlantic salmon and ensuring relevant enzymatic activity during the pre-digestion phase. The pH was subsequently neutralized by the addition of 1.0 M sodium hydroxide (NaOH) until it reached 6.0. The entire mixture was then autoclaved at 121 °C for 15 min. The first autoclaving step was included to help dissolve the fish feed in water, making it easier to filter Out solid particles before the second autoclaving ensured complete sterilization. After autoclaving, the fish meal was filtered through a 1 mm wet sieve to eliminate any solid particles that might obstruct the silicon tubing, after which it was subjected to a second round of autoclaving. The retained material was minimal (typically < 5 g per 2 L batch) and consistent across batches. For the experiment, each bioreactor was supplied with its own individually prepared batch of fish feed medium. The control diet contained standard feed, whereas the two experimental diets included 5% and 10% BEE added to the sterilized media. To ensure sterility, BEE was passed through a 0.2 µm sterile membrane filter under aseptic conditions.

### SalmoSim in vitro system setup.

**Fig. 1:**
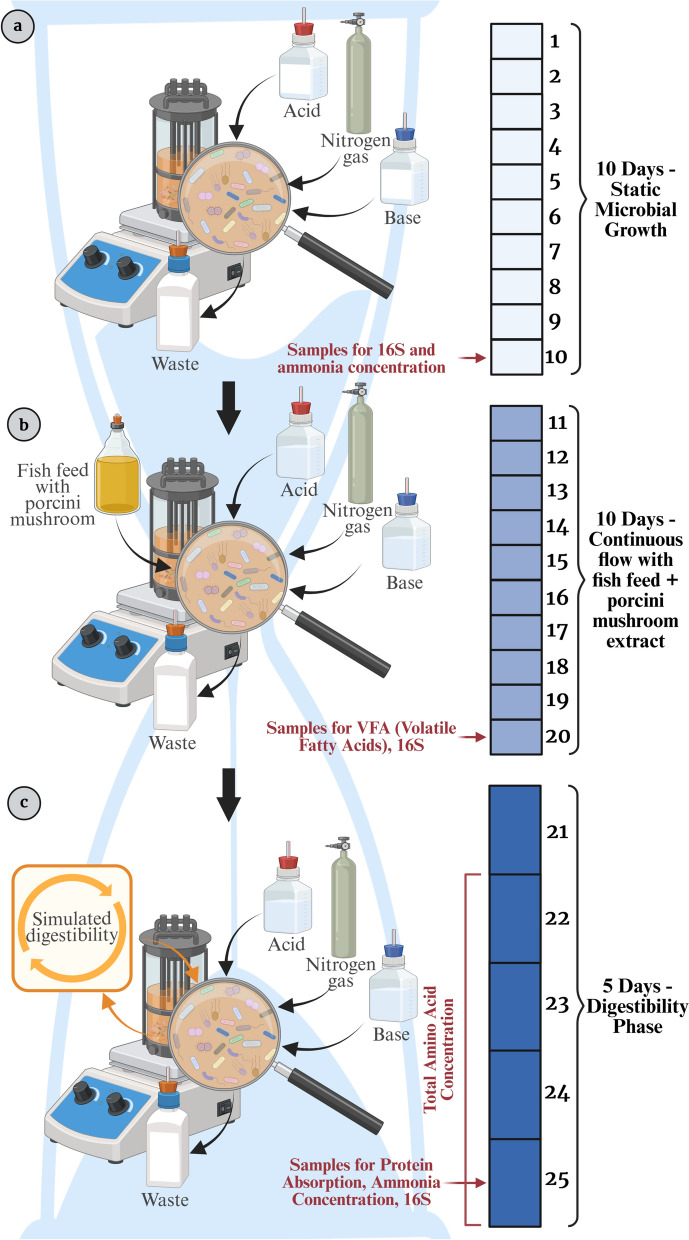
Experimental timeline and sampling strategy in the SalmoSim in vitro gut model. The system was run in three consecutive phases: **a** 10-day static microbial growth, **b** 10-day continuous feeding with fish feed and BEE, and **c** 5-day digestibility assessment

The study used nine custom-made double-jacketed glass bioreactors, each with a 700-mL capacity. Four 1 cm3 aquarium sponge filter pieces were used as substrates in each bioreactor to promote bacterial colonization. The Magnetic stirrer enabled continuous mixing. The whole apparatus, including the bioreactors and Magnetic beads, was autoclaved to guarantee sterility. Temperature and pH were monitored and continually adjusted. At the start of the experiment, each bioreactor received 400 mL of sterile feed medium. During the continuous flow phase, the fish feed was delivered at a rate of 200 mL per day. Simultaneously, the waste slurry was evacuated with peristaltic pumping to Maintain a consistent total volume of 400 mL per bioreactor (Fig. 1).

#### Note on system design and comparison to previous SalmoSim studies

Previous SalmoSim studies have employed a three-compartment, serial bioreactor configuration to simulate the stomach, pyloric caeca, and midgut [44]. In contrast, our experiment used nine independent single-compartment bioreactors operated in parallel, each seeded with the pyloric caecal microbiota from one of three individual Atlantic salmon. The pyloric caecum was chosen because it is the primary site of nutrient absorption in *Salmo salar* and reflects the overall gut microbial composition [50]. To simulate the stomach phase biochemically, the feed medium underwent acidification (pH 3.5) and enzymatic digestion with salmon-derived enzymes for 60 min**,** as detailed in the feed preparation protocol. This approach allowed us to mimic gastric digestion conditions without requiring a dedicated stomach bioreactor, streamlining the system while maintaining physiological relevance. Each of the three fish provided a distinct gut microbiota inoculum, which was used to seed three separate bioreactors, one for each dietary treatment (Control, 5% BEE, and 10% BEE).

### Inoculation and maintenance of bioreactors

One gram of microbiome gut mucus from the pyloric cecum sample was suspended in 1 mL of autoclaved 35 g/L Instant Ocean Sea Salt (Aquarium Systems, USA) solution and put into each bioreactor to introduce the microbiome. The source microbiome used here corresponds to the same pyloric caecal community that has been previously characterized in [51]. To ensure anaerobic conditions, dissolved oxygen was reduced by daily sparging with sterile nitrogen gas. The pH was Maintained at 7.0, to reflect the physiological conditions of the pyloric caecum in Atlantic salmon [44], while the temperature of the bioreactors was kept at 12 °C. Additionally, 0.5 g of salmon gut mucus solution and 1 ml of filtered bile obtained from the same individual fish were added daily.

### The experimental plan

The experimental plan consists of three distinct phases designed to investigate microbial growth, the effects of nutrient flow, and the digestibility of microbial communities.

#### Phase 1: Static microbial growth (Days 1–10)

The objective of Phase 1 was to create static microbial growth for 10 days to acquire baseline microbial populations. At the start of this phase, 400 mL of sterile feed medium was added to each bioreactor, but no additional nutrient input or waste outflow occurred throughout the rest of the phase. This approach was intended to provide a stable baseline for microbial populations.

On day 10, samples were obtained to assess the 16S rRNA gene sequences and ammonia levels. During the 10-day pre-treatment phase, bioreactors were fed batch-wise and maintained under static conditions (Fig. 1a). By the end of this phase, a visible mucus-like bacterial layer had formed on the vessel walls, indicating early biofilm development and microbial colonization.

#### Phase 2: Treatment phase with fish feed and BEE (days 11–20)

Phase 2 aimed to investigate the effects on BEE on microbial community composition and fermentative activity. In this setup, nutrient addition was facilitated through peristaltic pumps that continuously added fish feed and BEE to the system. Specifically, three bioreactors (B1, B2, and B3) served as the control group with no BEE added. Another three bioreactors (B4, B5, and B6) received a low dose of BEE (1 g per 20 g fish feed, equivalent to 5% of fish feed weight and 0.05% (w/v) in the total solution). The final three bioreactors (B7, B8, and B9) received a high dose of BEE (2 g per 20 g fish feed, equivalent to 10% of fish feed weight and 0.1% (w/v) in the total solution). This continuous nutrient flow was Maintained for 10 days. On Day 20, samples were collected from each bioreactor for downstream analysis. 10 mL of content was withdrawn for volatile fatty acid (VFA) analysis; samples were centrifuged at 10,000 × g for 10 min at 4 °C, and the supernatant was filtered through a 0.22 µm membrane filter (Fig. 1b). The pellet was retained for 16S rRNA gene sequencing. An additional 10 mL was used for ammonia concentration measurements using a commercial colorimetric assay kit, following the manufacturer’s instructions. For protein content determination, 20 mL of bioreactor content was dried at 50 °C for 24 h in a laboratory dryer, and the resulting dried mass was scraped, and stored for analysis.

#### Phase 3: Digestibility phase (Days 21–25)

The purpose of Phase 3 was to determine the predicted nitrogen digestibility under different experimental conditions (BEE 10%; BEE 5%; Control). Microfiltration of the bioreactor contents was performed using a 3.5 kDa pore Size membrane, and apparent digestibility was calculated alongside the analysis of small molecules, including amino acids. Phase 3 was started after 21 days because previous SalmoSim work [44] shows that by this point the microbial community composition stabilizes and closely resembles the original inoculum. The 5-day duration of Phase 3 was chosen to allow digestibility to be assessed under these stable conditions. On the final day of the experiment (Day 25), samples were collected for total amino acid analysis (1 mL in triplicate), while additional samples were taken following the same procedures as in Phase 2 for 16S rRNA gene sequencing, ammonia concentration, and crude protein content (Fig. 1c). Full methodological details of the microfiltration and digestibility analysis are provided in Supplementary File 1. A technical failure four days before the end of the experiment in one BEE 10% bioreactor reduced the sample Size to 26.

### Microbial DNA extraction and NGS library preparation

For 16S rRNA gene sequencing, the following DNA extraction and next-generation sequencing (NGS) library preparation protocols similar to those in [44, 50] were employed. In brief, for the samples collected from the SalmoSim system we used the QIAamp PowerFecal Pro DNA Kit—Stool/Gut DNA Extraction. Following extraction, the DNA was amplified with primers targeting the V1 region of the bacterial 16S rRNA gene under the following PCR conditions: initial denaturation at 95 °C for 10 min; 25 cycles of 95 °C for 30 s, 55 °C for 30 s, and 72 °C for 30 s; and a final elongation at 72 °C for 10 min. The primer sequences used were 27 F (5'-AGAGTTTGATCMTGGCTCAG-3') and 338R (5'-TGCTGCCTCCCGTAGGAGT-3'). The V1 region of the bacterial 16S rRNA gene was selected instead of the V4 region because it showed reduced contamination from salmon DNA, allowing for more accurate identification of the microbial community in the samples [51, 52].

A second round of PCR, which added external multiplex identifiers (barcodes), involved only six cycles with the same reaction conditions as the first round. The PCR products were then cleaned using Agencourt AMPure XP beads (Beckman Coulter, USA) according to the manufacturer’s protocol and gel-purified with the QIAquick Gel Extraction Kit (Qiagen, Valencia, CA, USA). The cleaned PCR products were pooled at a concentration of 10 nM and sent for sequencing via an Illumina MiSeq sequencer.

### 16S rRNA gene sequencing and microbial diversity analysis pipeline

For 16S rRNA sequencing analysis, we utilized the DADA2 pipeline [53], beginning with the import of raw sequencing data files in the FASTQ format. The samples were filtered and trimmed on the basis of quality scores, followed by error rate learning for both forward and reverse reads. We performed dereplication to collapse identical reads and then applied the DADA2 algorithm for sample inference. The forward and reverse reads were merged, and a sequence table was generated. Chimeric sequences were removed to ensure clean data, and taxonomic assignment was performed through the SILVA v138 reference database [54]. Finally, we tracked read retention through each step, saving the resulting sequence and taxonomy data for further analysis.

**Fig. 2:**
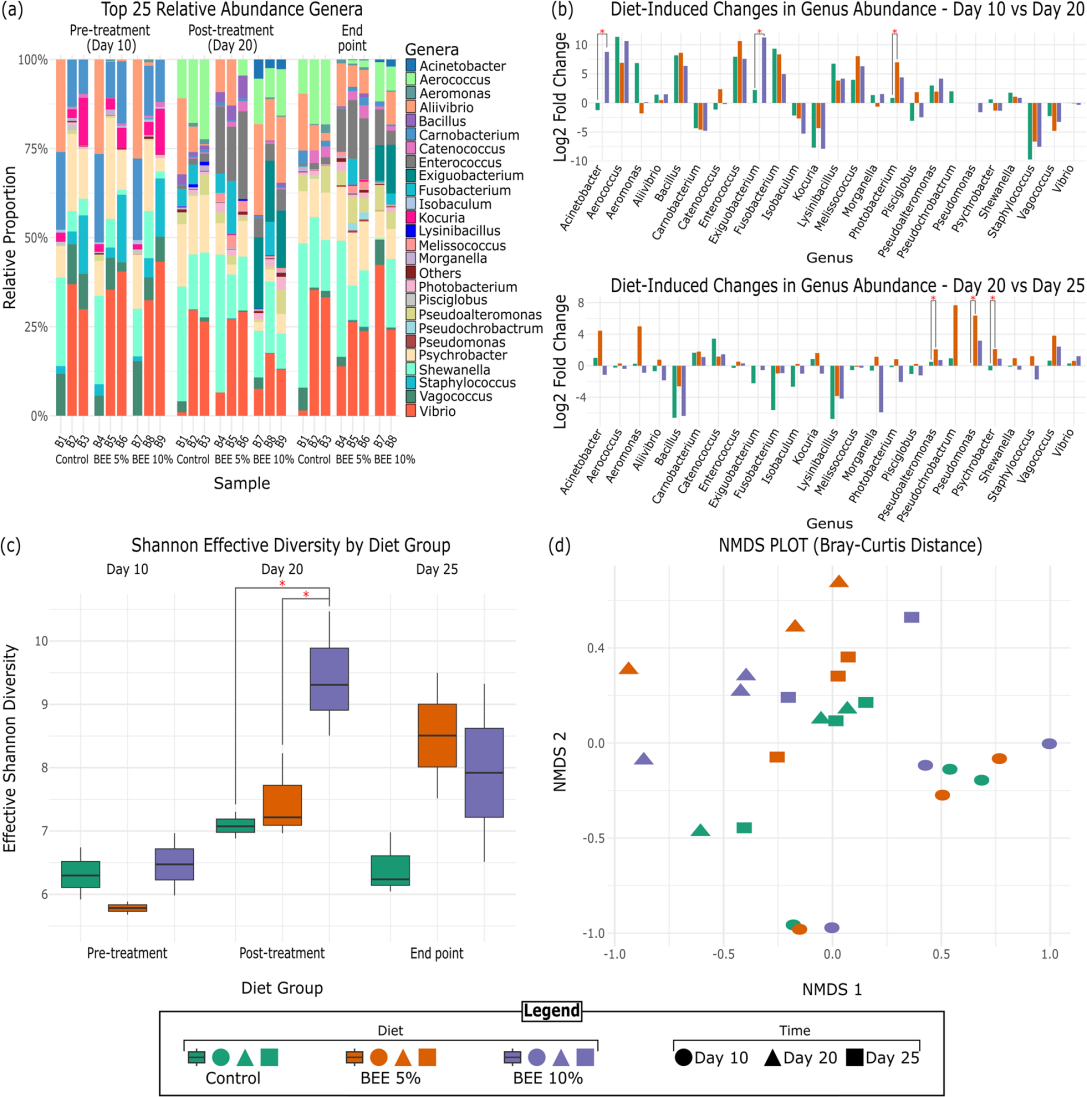
Microbial Community Shifts and Diversity in Response to BEE Supplementation. **a** Top 25 relative abundance genera: Relative abundance of the top 25 genera across Control, BEE 5%, and BEE 10% groups at Pre-treatment (Day 10), Post-treatment (Day 20), and End point (Day 25) **b** Log2-fold: Diet-induced changes between Day 10 and Day 20 (top) and between Day 20 and Day 25 (bottom) with red asterisks indicate significant differences in genus abundance between the diet groups. **c** Shannon effective diversity index with red asterisks indicating statistically significant differences between groups. **d** NMDS plot (Bray‒Curtis distance): Nonmetric multidimensional scaling (NMDS) plot illustrating beta diversity (stress = 0.17)

After the 16S rRNA gene data were processed with the DADA2 pipeline, the resulting data were analyzed in R. First, we loaded the sequence table and taxonomy data and then created a phyloseq [55] object to facilitate downstream analyses. The amplified sequence variant (ASV) table was extracted and transposed to match the taxonomy data. Both tables were merged, resulting in a combined abundance table that included taxonomic information alongside the corresponding sequence abundance data for each sample. To calculate relative abundance, we transformed the raw abundance data with the dplyr [56] and tidyr [57] Libraries, identifying the top 25 most abundant genera. Abundance was normalized by dividing each genus's abundance by the total sample abundance. A stacked bar plot, created with ggplot2 [58], was used to visualize the microbial community composition across samples, highlighting the relative proportions. To observe changes in abundance over time, we calculated log2-fold changes between time points for each genus and condition, utilizing the same R packages to quantify and compare microbial shifts. Additionally, we calculated the Shannon diversity index to assess microbial diversity across all time points and used the exponential of the Shannon index to calculate the Shannon effective diversity. To further evaluate microbial diversity and community structure, we performed rarefaction curves to assess sequencing depth and nonmetric multidimensional scaling (NMDS) using the vegan package [59] in R (Fig. 2).

### VFA measurement and analysis

**Fig. 3:**
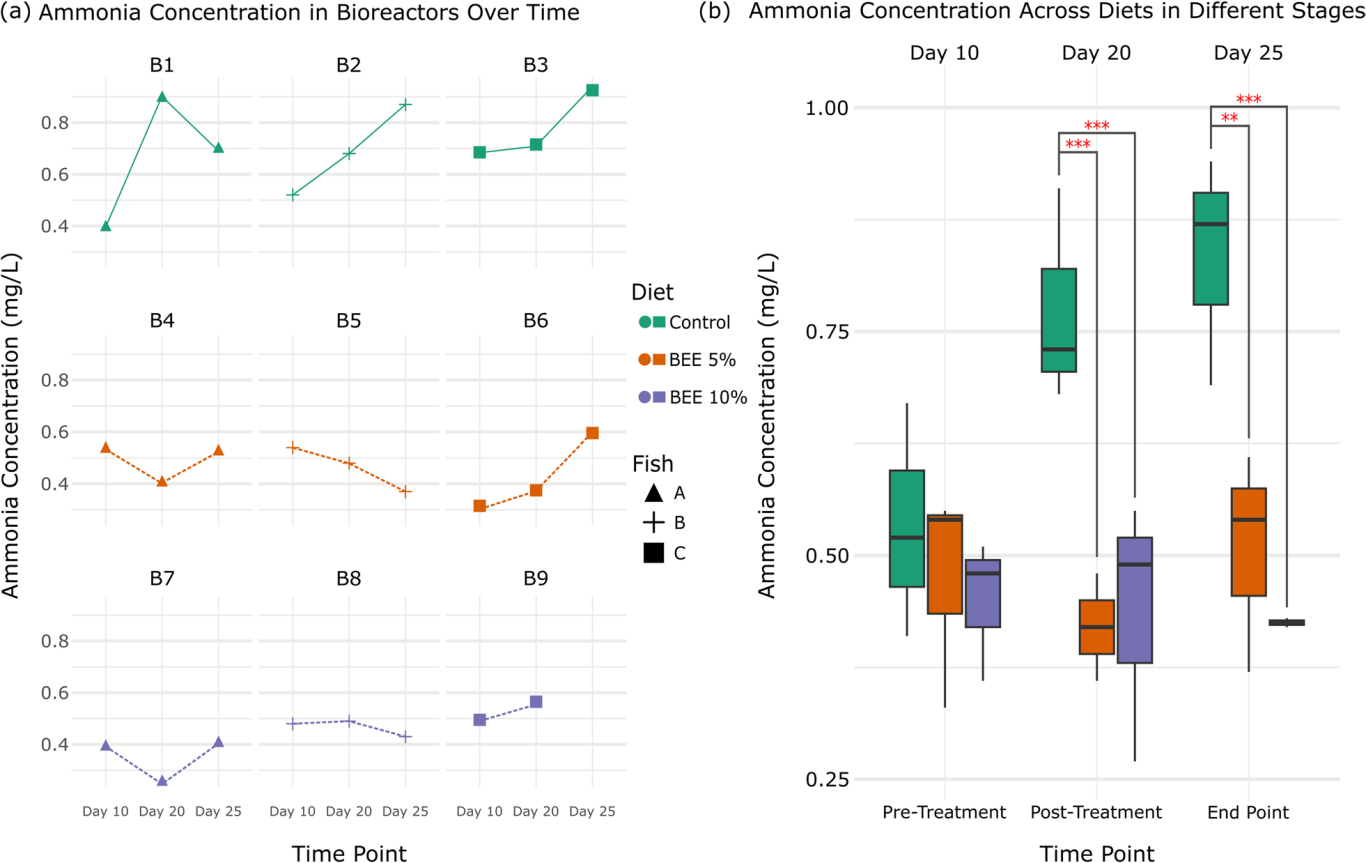
Ammonia concentration over time and across diets. **a** Ammonia concentration in bioreactors over time: Ammonia levels measured in bioreactors B1–B9 for the control, BEE 5%, and BEE 10% diets. **b** Ammonia concentration across diets at different stages: Box plots showing ammonia levels at different stages (Pre-treatment, Post-treatment, and End Point) across diets. Red asterisks indicate significant differences between groups (**p < 0.01, *p < 0.001)

Samples were collected in duplicate from each bioreactor on day 21 (Fig. 3), Marking the end of the continuous flow phase. The samples were then centrifuged at 10,000×g for 10 min to separate the supernatant, which was subsequently filtered Spin-X 0.22um cellulose acetate centrifuge tube filter (Costar®), snap frozen and kept at −80°C to prevent degradation of the VFAs.

The filtered and frozen supernatant samples were analyzed using gas chromatography–mass spectrometry at SUERC as previously described in [60]. Fatty acid concentrations were expressed in millimoles per liter (mM) of the aqueous phase (supernatant). For each bioreactor, duplicate measurements were averaged to obtain a single value per bioreactor. Subsequently, values from bioreactors representing the same diet were merged, and comparisons between different diet groups were conducted. These values reflect the direct concentrations in the liquid fraction obtained from the bioreactors.

#### Crude protein digestibility measurement and analysis

Crude protein digestibility was assessed for each bioreactor by measuring the nitrogen content in both the undigested and the Outgoing digested feed. This assessment was conducted via the Kjeldahl method, a standard procedure for determining nitrogen content. Crude protein was then calculated by applying a nitrogen-to-protein conversion factor of 6.25, which is standard for fish feed and microbial biomass.

The apparent digestibility coefficient (ADC) for crude protein was calculated through the following formula:

$${\text{ADC = ((Nitrogen\_CONC digesta (g/kg)/Nitrogen\_CONC feed (g/kg))}}*{100)}$$

This coefficient represents the proportion of nitrogen in the feed that was digested and absorbed, expressed as a percentage.

### Amino acid absorption measurement and analysis

Amino acid absorption was measured on the final day of the experiment, day 25, to estimate the amino acids absorbed by the salmon circulatory system during the digestibility phase. The quantification of amino acids was performed using a phthalaldehyde photometric assay following the method described in [61], with reference to a standard curve of alanine solutions to calculate absolute concentrations. Each unique sample was analyzed in technical triplicates and the average was calculated for each bioreactor sample. The resulting amino acid absorption data were subjected to statistical analysis to assess the effects of different concentrations of BEE extract on amino acid absorption across the various bioreactors and diets.

### Ammonia production measurement and analysis

Ammonia concentrations were measured using the Palintest Ammonia Test Kit (Palintest Ltd., UK), which is based on the indophenol blue method. In this reaction, ammonia reacts with alkaline salicylate in the presence of chlorine to form a green–blue indophenol complex. Reagents were supplied as Palintest Ammonia No. 2 tablets, and the reaction was conducted in 10 mL round test tubes (PT 595). Absorbance was measured using a Palintest photometer, and ammonia concentration was quantified using a standard curve generated from ammonium chloride (NH₄Cl) standards.

### Statistical analysis

All statistical analyses were performed using RStudio (version 2023.09.1). For microbiome data, alpha diversity indices (Shannon index and Shannon effective diversity) were compared between dietary treatments using one-way ANOVA followed by Tukey’s post hoc test [44]. Differences in community structure (beta diversity) between treatments and time points were assessed using Bray–Curtis distances and permutational multivariate analysis of variance (PERMANOVA, adonis function in the vegan package). Non-metric multidimensional scaling (NMDS) was used for ordination and visualization of beta-diversity patterns. Differential abundance at the genus level was tested using ANOVA (or Kruskal–Wallis when assumptions were not met) with Benjamini–Hochberg correction for multiple testing. One-way ANOVA followed by Tukey’s post hoc test [62] was used to assess differences between dietary treatments, with a significance threshold set at p < 0.05. For chemical analyses, differences in VFA concentrations and ammonia levels were evaluated across treatment groups and experimental phases. Significance was reported at p < 0.05, p < 0.01, and p < 0.001 where applicable.

## Results

To investigate the impact of BEE on the gut microbiota and nutritional potential of *Salmo salar* (Atlantic salmon), we evaluated microbial diversity, VFA synthesis, ammonia concentration, total amino acid levels, and crude protein digestibility using an in vitro SalmoSim model. The experiment utilized nine bioreactors running in parallel, with each assigned to one of three diet groups (Control, BEE 5%, and BEE 10%).

### Read filtering, merging, and taxonomic classification

A total of 1,441,133 raw reads were obtained from the 26 samples, with a median of 55,428 reads per sample. After filtering, 1,372,174 reads remained. The denoising step retained 1,280,181 forward reads and 1,318,852 reverse reads, which were subsequently merged into 1,115,093 paired reads. Chimeric sequences were removed, yielding 416,621 nonchimeric reads, corresponding to a 28.9% retention rate. Taxonomic classification was performed through the SILVA v138 database, providing the foundation for downstream ecological analyses.

### Microbial shifts and diversity analyses in response to BEE treatment

The relative abundance of microbial genera changed substantially across the three diet groups (Control, BEE 5%, and BEE 10%) at three time points: Pre-treatment (Day 10), Post-treatment (Day 20), and End Point (Day 25). Diversity and clustering analyses were based on all ASVs, while only the 25 most abundant microbial genera were visualized to compare their relative proportions across samples (Fig. 2a). The full 16S rRNA gene sequencing genus-level abundance data for each sample is provided in Supplementary Table 1.

In the Pre-treatment phase (Day 10), the genus *Vibrio* was notably absent in fish inoculum A (bioreactors B1, B4, and B7) across all the diet groups. Instead, genera such as *Psychrobacter*, and *Shewanella* dominated the microbial communities at this early point. *Vibrio* populations generally decreased at the end of the treatment phase fish B and C across all diet groups. Specifically, a slight reduction in relative abundance was noted in the BEE 10% treatment group compared to the BEE 5% group and the control, suggesting a potential suppressive effect at higher BEE concentrations. In contrast, the genus emerged and showed a modest increase in fish A, reflecting a differential response among fish and dietary treatments. By Day 20 (post-treatment), the genus *Vibrio* appeared in the microbial communities with a slight increase in relative abundance, particularly in the BEE-treated groups. Furthermore, we notice that *Carnobacterium* populations sharply decreased in all groups. By Day 25 (end point), *Vibrio* re-established itself as one of the dominant genera in both the control and BEE-treated groups.

Statistical analyses revealed significant microbial shifts among the treatment groups. *Acinetobacter* (p = 0.0053) and *Exiguobacterium* (p = 0.0098) showed Significant increases in the 10% BEE group compared to the control from Day 10 to Day 20 (Fig. 2b). *Photobacterium* also increased Significantly in the 5% BEE group compared to the control during the same period (p = 0.0057). Additionally, the abundances of *Pseudoalteromonas* (p = 0.0095), and *Psychrobacter* (p = 0.0101) were Significantly higher in the 5% BEE group compared to the control on Day 25 (Fig. 2b.).

### Diversity and clustering

The Shannon diversity index (Fig. 2c) showed a substantial increase in microbial diversity over time, particularly in the BEE 10% group. Significant changes were observed between Day 10 and Day 20 (p < 0.05), indicating that BEE supplementation positively influenced the gut microbiota diversity of Atlantic salmon.

The NMDS plot (Fig. 2d) illustrates beta diversity with the help of Bray–Curtis distances, revealing that microbiota clustering was initially influenced by individual fish (Pre-treatment). The stress value of 0.17 indicates a good fit of the ordination to the underlying data. By Day 20, the BEE-treated groups diverged from the control group, with the BEE 10% group showing the most pronounced separation. By Day 25, this separation persisted, reflecting sustained changes in microbial composition due to BEE supplementation, which was consistent with the increase in microbial diversity observed in the Shannon index.

### The effects of BEE on ammonia production in SalmoSim

Ammonia concentrations across the diet groups at Pre-treatment (Day 10), Post-treatment (Day 20), and End point (Day 25) were also compared. On Day 10 (Pre-treatment), ammonia concentrations were Similar across all diet groups, representing baseline levels prior to dietary intervention. By Day 20 (Post-treatment), a Significant increase in ammonia concentration was observed in the Control group compared with the BEE-treated groups. The Control group had a much higher median ammonia concentration, indicating that standard fish feed May promote ammonia accumulation, while BEE supplementation mitigated this effect. By Day 25 (End point), the ammonia levels in the Control group were still higher on average than in the BEE groups, though with increased variability across replicates. In contrast, the BEE 5% and BEE 10% groups presented more moderate and stable ammonia concentrations. Notably, the BEE 10% group presented the lowest median ammonia concentration by the end of the experiment. The statistical Significance of the reductions in ammonia levels in the BEE-treated groups compared with the control group is indicated by asterisks, with the BEE 10% group showing highly Significant differences at both day 20 and day 25 (p < 0.001).

### Volatile fatty acids (VFA) concentrations in response to BEE supplementation

**Fig. 4:**
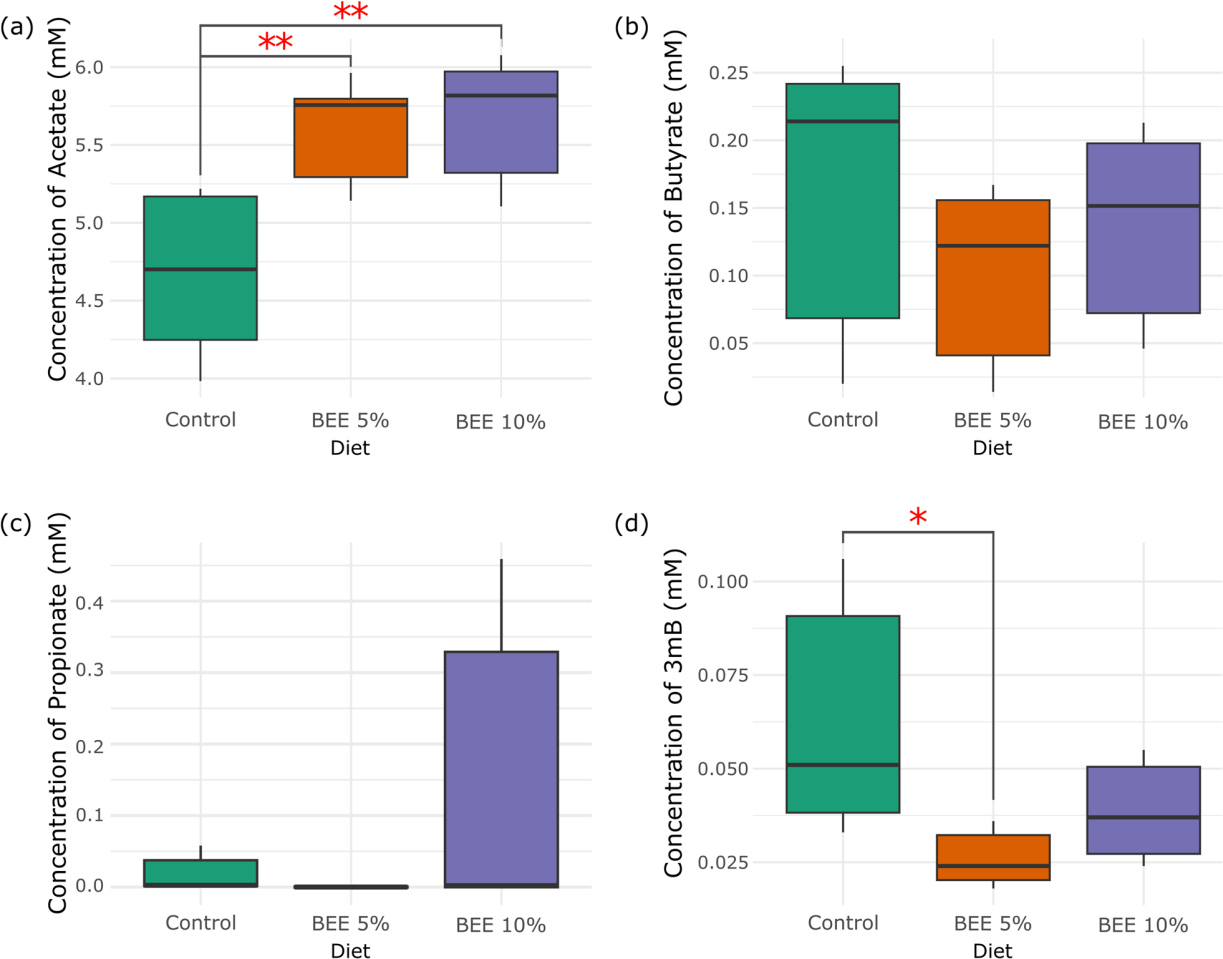
Comparison of SCFA concentrations according to diet. **a** Acetate, **b** butyrate, **c** propionate, and **d** 3-methylbutyrate (3mB) concentrations across diets (Control, BEE 5%, BEE 10%). Red asterisks indicate significant differences between groups (p < 0.01, p < 0.05)

VFA concentrations were measured on day 20, following the cessation of BEE treatment, to assess the effects of BEE supplementation on gut microbial metabolism. These concentrations were compared across three diet groups: Control, BEE 5%, and BEE 10%, providing insights into the impact of BEE on acetate, butyrate, propionate, and 3-methylbutyrate levels. Figure 4 presents the concentrations of various short-chain fatty acids across the different diet groups: Control, BEE 5%, and BEE 10%. Panel (a) shows that the acetate concentration was significantly greater in both the BEE groups than in the control group (p < 0.01), indicating increased acetate production with BEE supplementation.

Panel (b) shows that butyrate concentrations are highest in the control group, with reduced levels observed in the BEE 5% and BEE 10% groups. Panel (c) shows a numerical increase in propionate concentration in the BEE 10% group compared with the control and BEE 5% groups; however, this trend was not statistically Significant, Likely due to higher variability among bioreactors within the BEE 10% group. Panel (d) shows a Significant reduction in 3-methylbutyrate concentrations in the BEE 5% groups compared with those in the control group (p < 0.05).

### Dose-dependent improvements in amino acid absorption and protein utilization by BEE diets

At the end of the digestibility phase, we measured the absorption of total amino acids and the digestibility of crude protein across the different diet groups (Control, BEE 5%, and BEE 10%).

Panel (a) shows that amino acid absorption was highest in the 10% BEE group, with Significantly greater values than both the control and 5% BEE groups (p < 0.05). The 5% BEE group also showed increased absorption compared with the control, though to a lesser extent. Panel (b) displays the ADC of crude protein. Similarly, the BEE 10% group had the highest protein digestibility, with Significantly greater values than both the control and BEE 5% groups (p < 0.05). These results indicate that BEE supplementation improved both crude protein digestibility and amino acid absorption in a dose-dependent Manner. Compared with the control group, the 5% BEE group presented moderate improvements in protein digestibility and had the lowest digestibility coefficients. Additionally, we performed a Kjeldahl analysis on the BEE, revealing that the extract contained 9.56% crude protein, which likely contributed to the protein content in the BEE-supplemented diets.

## Discussion

This study aimed to evaluate the effects of supplementing the diet of *Salmo salar* with a prebiotic extract derived from *Boletus edulis*. Porcini mushrooms are highly rich in polysaccharides, particularly beta-glucans, which are well-recognized for their immune-modulating and prebiotic properties, enhancing gut health by promoting beneficial microbial fermentation in vertebrates [42, 43, 63, 64]. In aquaculture, mushroom-derived polysaccharides have been shown to positively impact fish health [39, 65, 66], yet the specific effects of *Boletus edulis* polysaccharides on the gut microbiota have not been explored until now. Our findings demonstrate that BEE supplementation modulates the gut microbiota of Atlantic salmon in a dose-dependent manner, contributing to shifts in microbial diversity, as shown in Fig. 2a, b. Importantly, this experiment was conducted in biological triplicate, with three individual Atlantic salmon contributing to the biological variation observed in the gut microbiota across the different diets. The pyloric ceca were selected for analysis as they represent the main site of nutrient absorption in *Salmo salar* [50].

Specifically, each fish’s microbiota was represented in three different bioreactors (e.g., B1, B4, and B7 for Fish A, each receiving a different diet: control, BEE 5%, and BEE 10%). It is worth highlighting that, in B1, B4, and B7, there was no presence of *Vibrio* in the pre-treatment stage, in contrast to other bioreactors where *Vibrio* was dominant. This could be attributed to the intrinsic differences in the baseline gut microbiota of Fish A, possibly influenced by factors such as individual variation in diet, environment, or health status prior to the experiment [20, 67, 68]. The absence of *Vibrio* in these bioreactors highlights the natural variability in microbial composition between individual fish. The storage of intestinal samples was carefully managed to minimize any potential alterations to microbial community composition. Nevertheless, we acknowledge that effects on microbial viability or low-abundance taxa cannot be entirely ruled out. Importantly, all samples across the different experimental groups underwent identical storage and handling procedures, ensuring that any potential storage-related effects would be uniformly distributed and unlikely to bias the observed results.

Overall, since dietary intervention had not yet begun, the microbiota of the same fish was more similar across bioreactors, leading to limited differentiation between the groups at this early stage. It was not until after the introduction of the experimental diets during the continuous flow phase that microbial shifts started to emerge, as the diets began to influence the microbiota composition. These similarities in the microbiota between bioreactors of the same fish are also reflected in Fig. 2d, where clustering patterns suggest that the microbiota on Day 10 is driven more by the individual fish.

Following the start of the continuous flow phase, where fish feed was introduced to Simulate a dynamic gut environment, Significant changes in microbial composition were observed, particularly between day 10 and day 20. During this phase, the bioreactors were supplemented with different diets, with the control group receiving only fish feed and the treatment groups receiving fish feed + 5% or 10% BEE. The increases in *Acinetobacter* and *Exiguobacterium* (Fig. 2b) in the BEE 10% group suggest that the addition of BEE creates a favorable environment for certain genera, potentially due to their exploitation of polysaccharide substrates [69, 70] present in *Boletus edulis*.

Furthermore, the microbial shifts observed between day 20 and day 25 might also have been influenced by the introduction of the digestibility phase, where continuous flow was stopped and the bioreactors returned to a static growth environment. During this period, microbial communities were likely to metabolize the remaining nutrients, including indigestible fibers and polysaccharides. The enrichment of genera such as *Pseudomonas* (p = 0.0007), *Pseudoalteromonas* (p = 0.0095), and *Psychrobacter* (p = 0.0101) in the BEE 5% group between day 20 and day 25 reflects their ability to thrive in nutrient-rich environments, likely benefiting from the breakdown of complex compounds provided by the fish feed and BEE supplementation. Remarkably, *Photobacterium* showed a Significant increase in the 5% BEE group between Day 10 and Day 20 **(**p = 0.0057**)**, suggesting that moderate BEE supplementation May create favorable conditions for its growth. However, this effect was not observed in the 10% BEE group, indicating a potential dose-dependent response. *Photobacterium, Pseudomonas, Pseudoalteromonas, and Psychrobacterare* are known to play a role in the degradation of organic matter [71–73], which is consistent with their increased abundance during the digestibility phase.

Interestingly, the influence of individual fish-origins on microbial composition remained apparent throughout the experiment. While diet-induced changes became more pronounced at later time points, particularly in the BEE 10% group, the individual microbiota composition remains an important factor driving variation, as shown in Fig. 2d. This aligns with previous studies showing that individual variability in the gut microbiota can influence dietary responses, even in controlled settings [74, 75].

On Day 20, following the completion of the treatment phase, the control diet, consisting solely of fish feed, resulted in a marked increase in the ammonia concentration, particularly in bioreactor B1 (Fig. 3a), where ammonia levels spiked significantly. This increase in ammonia could be attributed to the accumulation of nitrogenous waste products, as gut bacteria metabolize proteins and other nitrogen-rich compounds [76]. Interestingly, the sharp increase in B1 may reflect individual variation in the donor fish’s gut microbiota, as B1 was seeded with microbiota from Fish A, which differed notably from other inocula (e.g., absence of *Vibrio* and dominance of *Psychrobacter* and *Shewanella*). Such microbial differences could have contributed to elevated protein fermentation activity and increased ammonia production in this specific bioreactor. The elevated ammonia levels in the control group indicate that standard fish feed, without supplementation, may promote ammonia production, potentially leading to adverse effects on gut health and overall host well-being [77, 78].

A more diverse microbial community is typically associated with increased metabolic capacity and resilience [79, 80], which could help regulate nitrogen metabolism and prevent the accumulation of harmful byproducts such as ammonia. For example, the enrichment of genera such as *Acinetobacter* and *Exiguobacterium* in the BEE 10% group (p = 0.0053 and p = 0.0098, respectively) suggests that these bacteria may play a role in nitrogen cycling or ammonia detoxification [81–84], contributing to the observed reductions in ammonia concentration. Additionally, the presence of β-glucans (3.7 ± 0.5%), in the BEE diets likely shifted the microbial metabolism toward saccharolytic fermentation, reducing reliance on protein fermentation as an energy source. This shift would result in amino acids being redirected into microbial biomass rather than being deaminated for energy, thereby lowering ammonia production. Moreover, the microbial diversity observed in the BEE-treated groups likely contributed to this reduction in ammonia production. Higher microbial diversity is associated with greater functional redundancy, meaning that multiple bacterial species can carry out the same metabolic processes, reducing the risk of dysbiosis and the overproduction of harmful compounds [85, 86]. This is particularly evident in Fig. 2c, where the BEE 10% group showed the most distinct separation from the control group by day 25, reflecting sustained changes in microbial composition. In the absence of sufficient alternate substrates (i.e. BEE), the gut bacteria in the control group may have relied more on protein fermentation, leading to the production of ammonia and other harmful byproducts [87, 88]. The higher 3mB levels observed in the control group support this finding (Fig. 4d), as 3mB is a branched-chain fatty acid produced from the fermentation of the branched-chain amino acid leucine, derived from protein digestion [89]. In contrast, the lower 3mB levels in the BEE-treated groups suggest that BEE supplementation promotes fiber fermentation over protein fermentation, reducing the production of ammonia.

Ammonia is a well-known metabolic byproduct of protein digestion and microbial activity in the gut [90]. Elevated ammonia levels can disrupt gut health by promoting inflammation, damaging the epithelial barrier, and impairing nutrient absorption [77, 78]. In aquaculture, high ammonia concentrations in the gut or environment can lead to reduced growth rates, impaired immune function, and even increased mortality in fish [91, 92]. Therefore, the reduction in ammonia levels in the BEE-treated groups suggests a protective effect of BEE, potentially improving overall fish health by promoting a more balanced microbial community and reducing the burden of harmful metabolites [39–41].

The elevated acetate concentrations in the BEE 5% and BEE 10% groups compared with control suggest enhanced microbial fermentation of dietary fibers introduced through BEE supplementation. This pattern is consistent with observations from SalmoSim trials using the prebiotic Bio-Mos®, where acetate and other VFAs increased significantly in response to mannan-oligosaccharides (MOS) supplementation [51]. Acetate is one of the primary fermentation products of gut bacteria and serves as a precursor for other SCFAs, such as butyrate [93]. However, in the BEE-treated groups, the microbial shift toward acetate and propionate producing bacteria such as *Acinetobacter*, *Photobacterium*, and *Pseudomonas* may have downregulated the pathways that convert acetate into butyrate [94, 95], resulting in lower butyrate levels. This rebalancing of microbial fermentation dynamics, driven by the increased diversity observed in the BEE-treated groups, suggests that BEE promotes the growth of bacterial populations that favor acetate production, while reducing butyrate synthesis (Fig. 4a, b). Although only a minor, non-significant increase in propionate was detected in the 10% BEE group (Fig. 4c), this trend may reflect subtle shifts in microbial metabolism. In fish, SCFAs such as propionate and butyrate are increasingly recognized for their roles in host metabolism and immune regulation. For example, propionate may influence inflammatory signaling and energy utilization pathways [96, 97], whereas butyrate supports the maintenance of gut epithelial integrity and promotes immunological homeostasis [98, 99]. Thus, the apparent reduction in butyrate alongside stable or slightly elevated propionate levels could suggest a modest shift in SCFA-mediated host-microbe interactions, although further investigation is needed to determine its functional relevance.

**Fig. 5:**
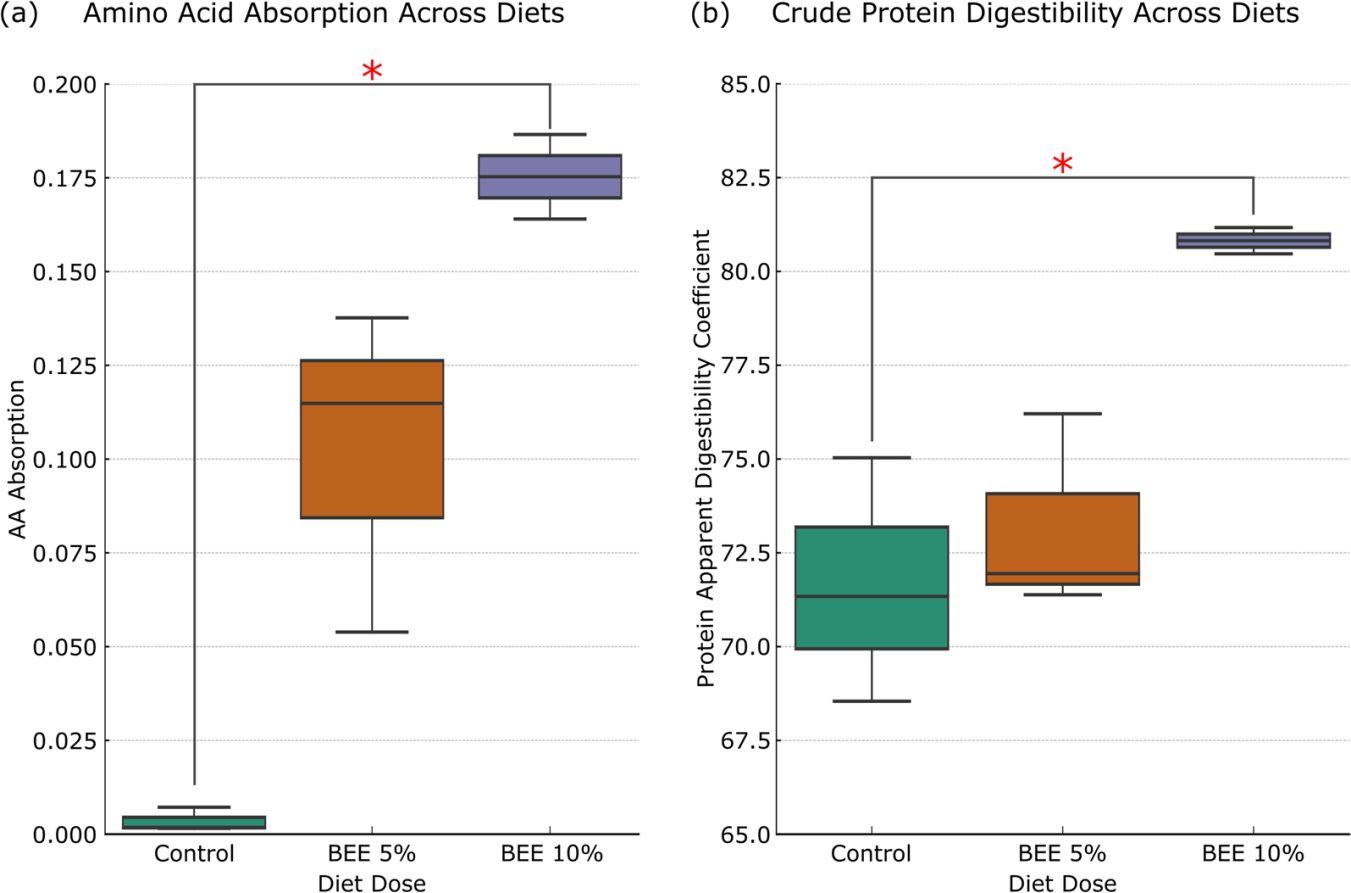
Amino acid absorption and crude protein digestibility across diets. **a** Total amino acid absorption and **b** crude protein digestibility for the control, BEE 5%, and BEE 10% diets at Day 25. Compared with the control group, the 10% BEE group presented significantly greater values (red asterisk, p < 0.05)

Furthermore, the use of microfiltration in the system allowed us to simulate microbial crude protein digestibility and absorption in a controlled environment. The enhanced absorption of amino acids and improved crude protein digestibility observed in the BEE-treated groups (Fig. 5), particularly the BEE 10% group, provide important insights into the broader microbial and metabolic shifts induced by BEE supplementation. These results align with the previously discussed changes in microbial diversity, SCFA production, and ammonia concentration, offering a comprehensive understanding of how BEE affects nutrient metabolism. Notably, despite 71% protein digestibility in the control group, amino acid absorption was undetectable (Fig. 5). This is likely to reflect microbial competition: while digestibility captures total nitrogen loss (including incorporation into microbial biomass), absorption only measures free amino acids. In fiber-deficient conditions, microbes may have outcompeted the host for amino acids, leaving little in the filtration. These findings suggest that BEE enhances not just digestion, but also nutrient bioavailability. The bioreactors Simulating the gut environment showed that 10% BEE supplementation supported a microbial ecosystem that favored fiber fermentation over protein fermentation [100], reducing microbial abstraction of crude protein/amino acids from the system, increasing their bioavailability to the fish and in doing so limiting the accumulation of harmful nitrogenous waste products such as ammonia (Fig. 3). Throughout all experimental phases, the pH was consistently Maintained at 7.0, reflecting the physiological conditions of the salmon pyloric caeca [44]. This pH stability has likely contributed to consistent microbial fermentation, supporting commensal taxa while reducing acidification-related stress. As pH is a key factor influencing ammonia generation and SCFA profiles [101, 102], maintaining this constant condition ensured that the observed metabolic shifts were primarily due to dietary treatment rather than fluctuations in pH.

While we quantified soluble amino acids, ammonia, and residual protein levels, we did not perform a complete nitrogen mass balance, therefore, the fate of total nitrogen inputs, including incorporation into microbial biomass or minor nitrogenous intermediates remains partially unresolved. Future studies could incorporate full nitrogen tracking to better capture microbial and host nitrogen utilization. However, we cannot discount the direct impact of BEE protein on the system (Kjeldahl analysis revealed that BEE contains 9.56% crude protein). The added protein from BEE likely contributed to the higher amino acid concentrations observed in the absorbed fraction (Fig. 5a), but it does not explain the improvement in crude protein digestibility (Fig. 5b), which reflects a relative increase in the proportion of protein digested. Given the digestibility of mushroom-derived proteins, it is plausible that some of these proteins were hydrolyzed, making amino acids available for incorporation into microbial biomass. Nevertheless, BEE contributed only ~ 2% of the total protein in the system, with the vast majority (~ 98%) derived from the fish feed (41.1% crude protein).

The results from the amino acid absorption and crude protein digestibility measurements also correlate with the increased microbial diversity observed in the BEE-treated groups. A more diverse gut microbiota is generally associated with enhanced metabolic versatility, allowing for more efficient nutrient processing [103, 104]. In addition, the control group exhibited both elevated ammonia levels (Fig. 3a) and reduced amino acid absorption (Fig. 5a) by Day 25, suggesting a correlation between inefficient amino acid utilization and increased nitrogenous waste production. This points toward enhanced microbial deamination of amino acids in the absence of alternative substrates, such as fiber, further reinforcing the Link between diet composition and microbial nitrogen metabolism. Moreover, the poorer performance of the control group in terms of amino acid absorption and protein digestibility, combined with higher ammonia and 3mB levels (Fig. 4d), further supports the hypothesis that BEE supplementation promotes a more beneficial gut microbial community.

In the control group, the gut microbiota May have relied more Heavily on protein fermentation, leading to the production of ammonia and BCFAs such as 3mB, both of which are associated with protein degradation and can have harmful effects on gut Health. In contrast, the BEE-treated groups, particularly the 10% group, appeared to shift microbial activity toward the fermentation of dietary fibers, promoting SCFA production and reducing reliance on protein fermentation, thus minimizing the production of harmful byproducts. Unlike commonly used prebiotics such as MOS [105] and fructo-oligosaccharides (FOS) [106], which are derived from yeast (*Saccharomyces cerevisiae*) [107] and plants (e.g., chicory root) [108], respectively, BEE is sourced from a wild ectomycorrhizal mushroom. Mushrooms differ from yeasts and plants in that they produce a wider array of complex bioactive compounds, including β-glucans and polyphenols with immunomodulatory and antioxidant potential [21, 109]. While *Boletus edulis* cannot currently be cultivated at scale due to its dependence on symbiotic tree associations, its effects may be attributed to general classes of compounds, particularly β-glucans, which have been shown to enhance gut microbial diversity and immune responses in fish [41, 110, 111]. This suggests that similar prebiotic benefits may be achievable using other, more cultivable mushroom species or extracts enriched in β-glucans and related functional metabolites. In addition, future studies using isolated or more rigorously characterized mushroom polysaccharide fractions, alongside in vivo trials, are needed to verify these preliminary findings and to explore potential immunological or physiological benefits for salmon health.

## Conclusions

The results of the present study revealed that including BEE in the diet of Atlantic salmon modifies the gut microbiota, increasing microbial diversity and encouraging favorable metabolic alterations. The increase in acetate and the descriptive trend of propionate in the BEE-treated groups suggests a shift toward improved fiber fermentation, and the observed decrease in ammonia levels underlines the ability of BEE to reduce nitrogenous waste build-up, which may have negative consequences for gut health. Furthermore, BEE supplementation increased amino acid absorption and crude protein digestibility, most likely through an effect on microbial composition and fermentation activity. These observations, combined with earlier evidence that mushroom-derived components can modulate fish health, underscore the importance of further research on specific mushroom polysaccharides.

## Supplementary Information

Additional file 1.

Additional file 2.

## Data Availability

The 16S rRNA sequencing data supporting the findings of this study have been deposited in the NCBI Sequence Read Archive (SRA) under BioProject accession number PRJNA1235220.
