# Supplementary material for: Modulatory effects of Boletus edulis on the gut microbiota in Atlantic salmon (Salmo salar) utilizing an artificial teleost gut model
Source: Anim Microbiome. 2025 Oct 27;7:111. doi: 10.1186/s42523-025-00469-x (PMC12560320; doi:10.1186/s42523-025-00469-x)
Supplement: Supplementary file 1 — Additional file 1. [file 42523_2025_469_MOESM1_ESM.docx]

Supplementary file

**Absorption and Digestibility Protocol**

For the digestibility phase (days 21-25), the addition of feed medium was stopped. Samples of feed medium were collected and stored at -20°C. The digestates were subjected to absorptive depletion by peristaltic pumping at a rate of approximately 150ml/min through 13cm of 22mm ID SnakeSkin^TM^ Dialysis Tubing with a 3.5K molecular weight cut-off against 250ml chilled artificial seawater as dialysate for 24hr. Upon completion of 24hrs of absorptive depletion, samples of the enriched absorbed dialysate were collected and frozen at -20 °C until analysis for amino acid levels. The enriched dialysate was renewed with naive chilled artificial seawater and then the absorptive depletion was resumed. This process of absorption and collection of digestible nutrients was performed for a total of four periods of 24 hrs, making 96 hrs total. The digestate and the feed medium were analysed by Kjeldahl method for measurement of crude protein digestibility.
